# The logic induced by effect algebras

**DOI:** 10.1007/s00500-020-05188-w

**Published:** 2020-07-26

**Authors:** Ivan Chajda, Radomír Halaš, Helmut Länger

**Affiliations:** 1 Department of Algebra and Geometry, Faculty of Science, Palacký University Olomouc, 17. listopadu 12, 771 46 Olomouc, Czech Republic; 2 Institute of Discrete Mathematics and Geometry, Faculty of Mathematics and Geoinformation, TU Wien, Wiedner Hauptstraße 8-10, 1040 Vienna, Austria

**Keywords:** Lattice effect algebra, Lattice effect implication algebra, Effect algebra, Effect implication algebra, Finite effect algebra, Gentzen system, Algebraic semantics, 03G12, 03G25, 06A11, 06F99

## Abstract

Effect algebras form an algebraic formalization of the logic of quantum mechanics. For lattice effect algebras ${\mathbf {E}}$, we investigate a natural implication and prove that the implication reduct of ${\mathbf {E}}$ is term equivalent to ${\mathbf {E}}$. Then, we present a simple axiom system in Gentzen style in order to axiomatize the logic induced by lattice effect algebras. For effect algebras which need not be lattice-ordered, we introduce a certain kind of implication which is everywhere defined but whose result need not be a single element. Then, we study effect implication algebras and prove the correspondence between these algebras and effect algebras satisfying the ascending chain condition. We present an axiom system in Gentzen style also for not necessarily lattice-ordered effect algebras and prove that it is an algebraic semantics for the logic induced by finite effect algebras.

## Introduction

Effect algebras were introduced by D. Foulis and M. K. Bennett (Foulis and Bennett 1994; see also Bennett and Foulis 1995 and Botur and Halaš 2009) as an algebraic axiomatization of the logic of quantum mechanics. For enabling deductions and derivations in this logic, it is necessary to introduce the connective implication. It is worth noticing that for lattice effect algebras this task was solved in a different way in Chajda et al. (2017). The problem is that though the binary operation of an effect algebra is only partial, implication should be defined everywhere. If the considered effect algebra is lattice-ordered, then implication is usually defined by $x\rightarrow y:=x'+(x\wedge y)$ and called *Sasaki implication*, see e.g., Borzooei et al. (2018), Chajda and Länger (2019), Foulis and Pulmannová (2012) and Rad et al. (2019). Properties of this implication were described in these papers, and a certain axiomatization of the corresponding logic in Gentzen style (see e.g., Blok and Pigozzi 1989) was derived in Rad et al. (2019). However, non-lattice-ordered effect algebras are more important than lattice-ordered ones. As shown in Chajda et al. (2009), any effect algebra can be completed to a *basic algebra* which is total. For commutative basic algebras, a Gentzen system was presented in Botur and Halaš (2009) and for basic algebras in Chajda (2015). This motivates us to find such an axiomatization also for not necessarily lattice-ordered effect algebras.

Another reason for introducing implication in effect algebras is to show that this connective is related to conjunction via (left) adjointness, and hence effect algebras can be considered as left residuated structures; see Chajda and Länger (2019), Chajda and Länger (submitted).

We should not direct the attention of the reader to the quantum logic aspect only but also to psychology, etc. (see Aerts 2009). The reason is that the logic of finite effect algebras cannot completely cover quantum mechanics, but it can help in other areas.

The paper is organized as follows: First, we introduce the *natural implication* instead of the Sasaki implication. Then, we define lattice effect implication algebras having this type of implication together with one constant as fundamental operations. We prove that lattice effect algebras and lattice effect implication algebras are term equivalent and in a natural one-to-one correspondence. Then, we derive an algebraic semantics for lattice effect implication algebras. In the second part of the paper, we extend our investigations to not necessarily lattice-ordered effect algebras satisfying the ascending chain condition, in particular to finite effect algebras. Also in this case, we characterize the operation of implication in a similar way as it was done in the lattice case. Finally, we provide an algebraic semantics of effect implication algebras.

For concepts and results concerning effect algebras, the reader is referred to the monograph by Dvurečenskij and Pulmannová (2000). The following definition and lemma are taken from Dvurečenskij and Pulmannová (2000).

### Definition 1.1

An *effect algebra* is a partial algebra ${\mathbf {E}}=(E,+,{}',0,1)$ of type (2, 1, 0, 0) where $(E,{}',0,1)$ is an algebra and $+$ is a partial operation satisfying the following conditions for all $x,y,z\in E$:

1. If $x+y$ is defined, then so is $y+x$ and $x+y=y+x$,
2. $(x+y)+z$ is defined if and only if so is $x+(y+z)$, and in this case $(x+y)+z=x+(y+z)$,
3. $x+y=1$ if and only if $y=x'$.
4. If $1+x$ is defined, then $x=0$.

On *E*, a binary relation $\le$ can be defined by

$$\begin{aligned} x\le y\quad \text { if there exists some }z\in E\text { with }x+z=y \end{aligned}$$

($x,y\in E$). Then, $(E,\le ,0,1)$ becomes a bounded poset and $\le$ is called the *induced order* of ${\mathbf {E}}$. If $(E,\le )$ is a lattice, then ${\mathbf {E}}$ is called a lattice effect algebra.

### Lemma 1.2

If $(E,+,{}',0,1)$ is an effect algebra, $\le$ its induced order and $a,b,c\in E$, then

(i) $$a\le b$$ implies $$b'\le a'$$,
(ii) $$a''=a$$,
(iii) $a+b$ is defined if and only if $a\le b'$,
(iv) If $a\le b$ and $b+c$ is defined, then $a+c$ is defined and $a+c\le b+c$,
(v) If $a+c$ and $b+c$ are defined, then $a+c\le b+c$ if and only if $a\le b$,
(vi) If $a\le b$, then $a+(a+b')'=b$ and $(b'+(b'+a)')'=a$,
(vii) $$a+0=a$$,
(viii) $$0'=1$$ and $$1'=0$$.

## Lattice effect implication algebras

For lattice effect algebras $(E,+,{}',0,1)$, the *Sasaki implication* (called also *material implication* in Blok and Pigozzi (1989)) was introduced as follows:

$$\begin{aligned} x\rightarrow y:=x'+(x\wedge y) \end{aligned}$$

for all $x,y\in E$. For our sake, we will consider this in the following way:

$$\begin{aligned} x\rightarrow y:=y+(x\vee y)'=y+(x'\wedge y') \end{aligned}$$

for all $x,y\in E$ and call this kind of implication *natural implication*. Note that the natural implication of *x* and *y* coincides with the Sasaki implication of $y'$ and $x'$. The reason for this choice is that it turns out that some computations become more feasible with this natural implication than with the Sasaki one.

In the rest of our paper, the implication investigated in lattice effect algebras will be the natural one.

### Theorem 2.1

Let $(E,+,{}',0,1)$ be a lattice effect algebra and $a,b,c\in E$. Then,

(i) $a\le b$ if and only if $a\rightarrow b=1$,
(ii) If $a\le b'$, then $a+b=a'\rightarrow b$,
(iii) If $a\ge b$, then $a\rightarrow b=a'+b$,
(iv) If $a\ge b$, then $a\rightarrow b=b'\rightarrow a'$,
(v) If $$a\le b$$, then $$b\rightarrow c\le a\rightarrow c$$.

### Proof

(i) The following are equivalent:
  $$\begin{aligned} a&\le b, \\ a\vee b&=b, \\ (a\vee b)'&=b', \\ b+(a\vee b)'&=1, \\ a\rightarrow b&=1. \end{aligned}$$
(ii) If $a\le b'$, then $a'\rightarrow b=b+(a'\vee b)'=b+a''=b+a=a+b$.
(iii) If $a\ge b$, then $a\rightarrow b=a''\rightarrow b=a'+b$.
(iv) If $a\ge b$, then $a\rightarrow b=a'+b=b+a'=b''+a'=b'\rightarrow a'$.
(v) If $$a\le b$$, then $$b\rightarrow c=c+(b\vee c)'\le c+(a\vee c)'=a\rightarrow c$$.

$$\square $$

Concerning the reduct restricted to $\rightarrow$ and $\vee$, we can prove the following.

### Theorem 2.2

Let $(E,+,{}',0,1)$ be a lattice effect algebra. Then, the following identities hold in $(E,\vee ,\rightarrow ,{}',0,1)$:

(i) $$x\rightarrow 0\approx x'$$,
(ii) $$1\rightarrow x\approx x$$,
(iii) $$x\rightarrow (y\rightarrow x)\approx 1$$,
(iv) $$(x\rightarrow y)\rightarrow y\approx x\vee y$$,
(v) $$((x\rightarrow y)\rightarrow y)\rightarrow y\approx x\rightarrow y$$,
(vi) $$x\rightarrow ((x\rightarrow y)\rightarrow y)\approx 1$$,
(vii) $$y\rightarrow ((x\rightarrow y)\rightarrow y)\approx 1$$,
(viii) $$y'\rightarrow ((x\rightarrow y)\rightarrow y)'\approx x\rightarrow y$$.

### Proof

We have

(i) $$x\rightarrow 0\approx 0+(x\vee 0)'\approx x'$$,
(ii) $$1\rightarrow x\approx x+(1\vee x)'\approx x$$,
(iii) $$x\le x+(y\vee x)'=y\rightarrow x$$,
(iv) $(x\rightarrow y)\rightarrow y\approx y+((y+(x\vee y)')\vee y)'\approx y+(y+(x\vee y)')'\approx x\vee y$ according to (vi) of Lemma 1.2,
(v) $((x\rightarrow y)\rightarrow y)\rightarrow y\approx (x\rightarrow y)\vee y\approx x\rightarrow y$ according to (i) of Theorem 2.1 and (iii),
(vi) $x\le x\vee y=(x\rightarrow y)\rightarrow y$ according to (iv),
(vii) $y\le x\vee y=(x\rightarrow y)\rightarrow y$ according to (iv),
(viii) $y'\rightarrow ((x\rightarrow y)\rightarrow y)'\approx y'\rightarrow (x\vee y)'\approx y''+(x\vee y)'\approx y+(x\vee y)'\approx x\rightarrow y$ according to (iv), to (iii) of Theorem 2.1 and to (ii) of Lemma 1.2.

$$\square $$

Summarizing the above properties, we can introduce an alter ego of a lattice effect algebra, i.e., its implication version.

### Definition 2.3

A *lattice effect implication algebra* is an algebra $(I,\rightarrow ,0)$ of type (2, 0) satisfying the following conditions for all $x,y,z\in I$ (we abbreviate $x\rightarrow 0$ by $x'$ and $0'$ by 1):

(i) $$0\rightarrow x\approx x\rightarrow x\approx x\rightarrow 1\approx 1$$,
(ii) If $x\rightarrow y=y\rightarrow x=1$, then $x=y$,
(iii) If $x\rightarrow y=y\rightarrow z=1$, then $x\rightarrow z=1$,
(iv) If $x\rightarrow y=1$, then $y'\rightarrow x'=1$,
(v) $$x''\approx x$$,
(vi) $$x\rightarrow ((x\rightarrow y)\rightarrow y)\approx 1$$,
(vii) $$y\rightarrow ((x\rightarrow y)\rightarrow y)\approx 1$$,
(viii) If $x\rightarrow z=y\rightarrow z=1$, then $((x\rightarrow y)\rightarrow y)\rightarrow z=1$,
(ix) If $x\rightarrow y=1$, then $y\rightarrow x=x'\rightarrow y'$,
(x) $x\rightarrow y'=(x'\rightarrow y)\rightarrow z'=1$ if and only if $y\rightarrow z'=x\rightarrow (y'\rightarrow z)'=1$, and in this case $(x'\rightarrow y)'\rightarrow z=x'\rightarrow (y'\rightarrow z)$,
(xi) $$y'\rightarrow ((x\rightarrow y)\rightarrow y)'\approx x\rightarrow y$$,
(xii) $$x\rightarrow (y\rightarrow x)\approx 1$$.

In order to show the correspondence between a lattice effect algebra and the implication version, we state and prove the following three theorems.

### Theorem 2.4

Let ${\mathbf {E}}=(E,+,{}',0,1)$ be a lattice effect algebra with induced order $\le$ and lattice operations $\vee$ and $\wedge$ and put

$$\begin{aligned} x\rightarrow y:=y+(x\vee y)' \end{aligned}$$

for all $x,y\in E$. Then, ${{\mathbb {I}}}{\mathbb {L}}({\mathbf {E}}):=(E,\rightarrow ,0)$ is a lattice effect implication algebra where

$$\begin{aligned}&x\le y\text { if and only if }x\rightarrow y=1, \\&x\vee y\approx (x\rightarrow y)\rightarrow y\text { and }x\wedge y\approx (x'\vee y')'. \end{aligned}$$

for all $$x,y\in E$$.

### Proof

The proof follows from Theorems 2.1 and 2.2. More precisely, (i)–(iii) follow from the fact that $(E,\le ,0,1)$ is a bounded poset, (iv) follows from the fact that $'$ is antitone on $(E,\le )$, (v) from the fact that $'$ is an involution, (vi) and (vii) follow from the fact that $x\vee y$ is an upper bound of *x* and *y*, (viii) follows from the fact that $x\vee y$ is less than or equal to every upper bound of *x* and *y*, (ix) from (E1), (x) from (E2), (xi) from (viii) of Theorem 2.2 and (xii) from (iii) of Theorem 2.2. $\square$

### Theorem 2.5

Let ${\mathbf {I}}=(I,\rightarrow ,0)$ be a lattice effect implication algebra and put

$$\begin{aligned} x'&:=x\rightarrow 0, \\ 1&:=0', \\ x+y&:=x'\rightarrow y\text { if and only if }x\rightarrow y'=1 \end{aligned}$$

for all $x,y\in E$. Then, ${\mathbb {E}}{\mathbb {L}}({\mathbf {I}}):=(I,+,{}',0,1)$ is a lattice effect algebra with induced order $\le$ and lattice operations $\vee$ and $\wedge$ where

$$\begin{aligned}&x\le y\text { if and only if }x\rightarrow y=1, \\&x\vee y\approx (x\rightarrow y)\rightarrow y\text { and }x\wedge y\approx (x'\vee y')'. \end{aligned}$$

### Proof

Let $$a,b,c\in I$$. Define

$$\begin{aligned} x\le y\text { if and only if }x\rightarrow y=1 \end{aligned}$$

for all $x,y\in I$. Then, due to (i)–(v) of Definition 2.3, $(I,\le ,{}',0,1)$ is a bounded poset with an antitone involution. If $a+b$ is defined, then $a\le b'$ and hence $b\le a'$, i.e., $b+a$ is defined and

$$\begin{aligned} a+b=a'\rightarrow b=b'\rightarrow a=b+a \end{aligned}$$

according to (ix) of Definition 2.3. This shows (E1). Now, $a+b$ is defined if and only if $a\le b'$, and in this case $a+b=a'\rightarrow b$. If $a+b$ is defined, then $(a+b)+c$ is defined if and only if $a+b\le c'$, and in this case

$$\begin{aligned} (a+b)+c=(a+b)'\rightarrow c=(a'\rightarrow b)'\rightarrow c. \end{aligned}$$

Hence, $(a+b)+c$ is defined if and only if both $a\le b'$ and $a'\rightarrow b\le c'$. On the other hand, $b+c$ is defined if and only if $b\le c'$, and in this case $b+c=b'\rightarrow c$. If $b+c$ is defined, then $a+(b+c)$ is defined if and only if $a\le (b+c)'$, and in this case

$$\begin{aligned} a+(b+c)=a'\rightarrow (b+c)=a'\rightarrow (b'\rightarrow c). \end{aligned}$$

Hence, $a+(b+c)$ is defined if and only if both $b\le c'$ and $a\le (b'\rightarrow c)'$. According to (x) of Definition 2.3, (E2) holds. Now, the following are equivalent:

$$\begin{aligned}&a+b=1, \\&a\le b'\text { and }a'\rightarrow b=1, \\&b\le a'\text { and }a'\le b, \\&b=a'. \end{aligned}$$

This shows (E3). If $1+a$ is defined, then $1\le a'$ whence $a'=1$, i.e., $a=0$ showing (E4). Hence, ${\mathbb {E}}{\mathbb {L}}({\mathbf {I}})$ is an effect algebra. According to (vi)–(viii) of Definition 2.3, we have

$$\begin{aligned} x\vee y\approx (x\rightarrow y)\rightarrow y, \end{aligned}$$

and because $'$ is an antitone involution on $(I,\le )$ we have

$$\begin{aligned} x\wedge y\approx (x'\vee y')'. \end{aligned}$$

If $a\le b$, then $a\rightarrow (b\rightarrow a)''=a\rightarrow (b\rightarrow a)=1$ according to (xii) and

$$\begin{aligned} a+(b\rightarrow a)'&=a'\rightarrow (b\rightarrow a)' \\&=(b\rightarrow a)''\rightarrow (a'\vee (b\rightarrow a)')' \\&=(b\rightarrow a)\rightarrow a'' \\&=(b\rightarrow a)\rightarrow a=b\vee a=b \end{aligned}$$

according to (xi). If, conversely, $a+c=b$, then $a\rightarrow c'=1$ and $a'\rightarrow c=b$ and hence

$$\begin{aligned} a\le c'\rightarrow a=c'\rightarrow a''=c'\rightarrow (a'\vee c)'=a'\rightarrow c=b \end{aligned}$$

according to (xii) and (xi). This shows that induced order of ${\mathbb {E}}{\mathbb {L}}({\mathbf {I}})$ coincides with $\le$. $\square$

### Theorem 2.6

The correspondence between lattice effect algebras and lattice effect implication algebras described by Theorems 2.4 and 2.5 is one-to-one.

### Proof

Let ${\mathbf {E}}=(E,+,{}',0,1)$ be a lattice effect algebra, put ${\mathbb {I}}{\mathbb {L}}({\mathbf {E}})=(E,\rightarrow ,0)$ and ${\mathbb {E}}{\mathbb {L}}({\mathbb {I}}{\mathbb {L}}({\mathbf {E}}))=(E,\oplus ,{}^*,0,e)$ and let $a,b\in E$. Then, $a\oplus b$ is defined if and only if so is $a+b$ since both are equivalent to $a\le b'$, and in this case

$$\begin{aligned} a\oplus b=a'\rightarrow b=a+b \end{aligned}$$

according to Theorem 2.1. Moreover,

$$\begin{aligned} a^*=a\rightarrow 0=a' \end{aligned}$$

according to Theorem 2.2, and

$$\begin{aligned} e=0\rightarrow 0=0'=1 \end{aligned}$$

showing ${\mathbb {E}}{\mathbb {L}}({\mathbb {I}}{\mathbb {L}}({\mathbf {E}}))={\mathbf {E}}$. Conversely, let ${\mathbf {I}}=(I,\rightarrow ,0)$ be a lattice effect implication algebra, put ${\mathbb {E}}{\mathbb {L}}({\mathbf {I}})=(I,+,{}',0,1)$ and ${\mathbb {I}}{\mathbb {L}}({\mathbb {E}}{\mathbb {L}}({\mathbf {I}}))=(I,\Rightarrow ,0)$ and let $c,d\in I$. Then,

$$\begin{aligned} c\Rightarrow d=d+(c\vee d)'=d'\rightarrow ((c\rightarrow d)\rightarrow d)'=c\rightarrow d \end{aligned}$$

according to (xi). This shows ${\mathbb {I}}{\mathbb {L}}({\mathbb {E}}{\mathbb {L}}({\mathbf {I}}))={\mathbf {I}}$. $\square$

## Axioms and rules for the logic of lattice effect algebras

By a propositional language, we understand some set ${\mathcal {L}}$ of propositional connectives. The arity of a propositional connective $c\in {\mathcal {L}}$ is called the rank of *c*. The ${\mathcal {L}}$-formulas are built in the usual way from the propositional variables using the connectives of ${\mathcal {L}}$. We denote the set of all ${\mathcal {L}}$-formulas by ${\mathcal {F}}m_{{\mathcal {L}}}$ or ${\mathcal {F}}m$ in brief when the language ${\mathcal {L}}$ is clear from the context.

By a logic in ${\mathcal {L}}$, we mean a standard deductive system over ${\mathcal {L}}$ or, equivalently, its consequence relation $\vdash _{{\mathcal {L}}}$ (see Blok and Pigozzi 1989 for details). We shall use the notation $\vdash$ whenever there is no danger of confusion.

Let us mention that a system of axioms and rules for the propositional logic induced by lattice effect algebras was already presented in Rad et al. (2019). However, that system is rather complicated because it consists of five axioms and ten derivation rules. In what follows, we present another system having only three axioms and five rules. Moreover, three of these rules, namely Modus Ponens, Suffixing and Weak Prefixing, are common in many propositional calculi.

By the propositional logic $L_{\mathrm{LEA}}$ in a language ${\mathcal {L}}=\{\rightarrow ,0\}$ ($\rightarrow$ is of rank 2, 0 is of rank 0), we understand a consequence relation $\vdash _{\mathrm{LEA}}$ (or $\vdash$, in brief) satisfying the axioms

1. $$\vdash \varphi \rightarrow (\psi \rightarrow \varphi )$$,
2. $$\vdash ((\varphi \rightarrow \psi )\rightarrow \psi )\rightarrow ((\psi \rightarrow \varphi )\rightarrow \varphi )$$,
3. $$\vdash 0\rightarrow \varphi $$

and the rules

(MP) $$\varphi ,\varphi \rightarrow \psi \vdash \psi $$,
(Sf) $$\varphi \rightarrow \psi \vdash (\psi \rightarrow \chi )\rightarrow (\varphi \rightarrow \chi )$$,
(WPf) $$\varphi \rightarrow \psi ,\psi \rightarrow \varphi \vdash (\chi \rightarrow \varphi )\rightarrow (\chi \rightarrow \psi )$$,
(R1) $$\varphi \rightarrow \psi \vdash (\lnot \varphi \rightarrow \lnot \psi )\rightarrow (\psi \rightarrow \varphi )$$,
(R2) $$\varphi \rightarrow \lnot \psi ,(\lnot \varphi \rightarrow \psi )\rightarrow \lnot \chi \vdash (\lnot (\lnot \varphi \rightarrow \psi )\rightarrow \chi )\rightarrow (\lnot \varphi \rightarrow (\lnot \psi \rightarrow \chi ))$$,

where $$\lnot \varphi :=\varphi \rightarrow 0$$ and $$1:=\lnot 0=0\rightarrow 0$$.

The axiom system (A1)–(A3) with the derivation rules (MP)–(R2) will be referred to as *axiom system* ${\mathbf {A}}$.

As usual, for $\Gamma \cup \{\varphi ,\psi \}\subseteq {\mathcal {F}}m$, the biconditional $\Gamma \vdash \varphi \leftrightarrow \psi$ is an abbreviation for $\Gamma \vdash \varphi \rightarrow \psi$ and $\Gamma \vdash \psi \rightarrow \varphi$.

In order to show that the system ${\mathbf {A}}$ is really an axiom system in Gentzen style for lattice effect algebras, we prove the following important properties.

### Theorem 3.1

In the propositional logic $L_{\mathrm{LEA}}$, the following are provable:

- $$\varphi \rightarrow \psi ,\psi \rightarrow \chi \vdash \varphi \rightarrow \chi $$,
- $$\vdash \varphi \rightarrow 1$$,
- $$\vdash \varphi \rightarrow \varphi $$,
- $$\vdash \psi \rightarrow (\psi \vee \varphi )$$ where $$\psi \vee \varphi :=(\psi \rightarrow \varphi )\rightarrow \varphi $$,
- $$\vdash \lnot \lnot \varphi \leftrightarrow \varphi $$,
- $$\varphi \rightarrow \psi \vdash \lnot \varphi \rightarrow \lnot \psi \leftrightarrow \psi \rightarrow \varphi $$,
- $$\vdash \psi \rightarrow (\varphi \vee \psi )$$ and $$\vdash (\varphi \vee \psi )\leftrightarrow (\psi \vee \varphi )$$,
- $$\varphi \rightarrow \psi ,\chi \rightarrow \psi \vdash (\varphi \vee \chi )\rightarrow \psi $$,
- $$\vdash \varphi \rightarrow \psi \leftrightarrow (\varphi \vee \psi )\rightarrow \psi $$,
- $$\vdash \lnot \varphi \rightarrow \lnot (\psi \vee \varphi )\leftrightarrow \psi \rightarrow \varphi $$.

### Proof

- This follows from (Sf) and (MP).
- According to (A1),
  $$\begin{aligned} \vdash 1\rightarrow (\varphi \rightarrow 1) \end{aligned}$$
  and according to (A3)
  $$\begin{aligned} \vdash 0\rightarrow 0, \end{aligned}$$
  i.e.,
  $$\begin{aligned} \vdash 1. \end{aligned}$$
  Thus, applying (MP) we conclude
  $$\begin{aligned} \vdash \varphi \rightarrow 1. \end{aligned}$$
- According to (A1),
  $$\begin{aligned}&\vdash \psi \rightarrow ((\varphi \rightarrow \psi )\rightarrow \psi ), \\&\vdash \varphi \rightarrow (\psi \rightarrow \varphi ), \end{aligned}$$
  thus by (Sf),
  $$\begin{aligned} \vdash ((\psi \rightarrow \varphi )\rightarrow \varphi )\rightarrow (\varphi \rightarrow \varphi ). \end{aligned}$$
  Applying (A2) and (Sf), we have
  $$\begin{aligned}&\vdash (((\psi \rightarrow \varphi )\rightarrow \varphi ) \rightarrow (\varphi \rightarrow \varphi )) \\&\quad \rightarrow (((\varphi \rightarrow \psi )\rightarrow \psi )\rightarrow (\varphi \rightarrow \varphi )). \end{aligned}$$
  Now, (MP) yields
  $$\begin{aligned} \vdash ((\varphi \rightarrow \psi )\rightarrow \psi )\rightarrow (\varphi \rightarrow \varphi ). \end{aligned}$$
  Hence,
  $$\begin{aligned} \vdash \psi \rightarrow (\varphi \rightarrow \varphi ) \end{aligned}$$
  by (a). Substituting any provable formula for $\psi$ within the last formula yields
  $$\begin{aligned} \vdash \varphi \rightarrow \varphi \end{aligned}$$
  according to (MP).
- We have
  $$\begin{aligned} \vdash \psi \rightarrow (\varphi \vee \psi ) \end{aligned}$$
  according to (A1) and
  $$\begin{aligned} \vdash (\varphi \vee \psi )\rightarrow (\psi \vee \varphi ) \end{aligned}$$
  according to (A2), and hence
  $$\begin{aligned} \vdash \psi \rightarrow (\psi \vee \varphi ) \end{aligned}$$
  by (a).
- We have
  $$\begin{aligned} \vdash \lnot \lnot \varphi \rightarrow (0\vee \varphi ) \end{aligned}$$
  according to (A2) and
  $$\begin{aligned} \vdash (0\vee \varphi )\rightarrow \varphi \end{aligned}$$
  according to (A3), (d) and (MP). This shows
  $$\begin{aligned} \vdash \lnot \lnot \varphi \rightarrow \varphi \end{aligned}$$
  by (a). Conversely,
  $$\begin{aligned} \vdash \varphi \rightarrow \lnot \lnot \varphi \end{aligned}$$
  according to (d).
- According to (R1), we have
  $$\begin{aligned} \varphi \rightarrow \psi \vdash (\lnot \varphi \rightarrow \lnot \psi )\rightarrow (\psi \rightarrow \varphi ). \end{aligned}$$
  Moreover,
  $$\begin{aligned} \vdash (\psi \rightarrow \varphi )\rightarrow (\lnot \varphi \rightarrow \lnot \psi ) \end{aligned}$$
  according to (Sf).
- We have
  $$\begin{aligned} \vdash \psi \rightarrow (\varphi \vee \psi ) \end{aligned}$$
  according to (A1). The rest follows from (A2).
- Applying (Sf) twice, we obtain
  $$\begin{aligned} \varphi \rightarrow \psi&\vdash (\psi \rightarrow \chi )\rightarrow (\varphi \rightarrow \chi ), \\ (\psi \rightarrow \chi )\rightarrow (\varphi \rightarrow \chi )&\vdash (\varphi \vee \chi )\rightarrow (\psi \vee \chi ) \end{aligned}$$
  and hence
  $$\begin{aligned} \varphi \rightarrow \psi \vdash (\varphi \vee \chi )\rightarrow (\psi \vee \chi ) \end{aligned}$$
  by (a). But
  $$\begin{aligned} \vdash (\psi \vee \chi )\rightarrow (\chi \vee \psi ) \end{aligned}$$
  according to (A2). Now,
  $$\begin{aligned} \chi \rightarrow \psi \vdash (\chi \vee \psi )\rightarrow \psi \end{aligned}$$
  according to (d). Hence, (h) follows from (a).
- We have
  $$\begin{aligned} \vdash (\varphi \rightarrow \psi )\rightarrow ((\varphi \vee \psi )\rightarrow \psi ) \end{aligned}$$
  according to (d). Conversely,
  $$\begin{aligned} \vdash ((\varphi \vee \psi )\rightarrow \psi )\rightarrow (\psi \vee (\varphi \rightarrow \psi )) \end{aligned}$$
  according to (A2). Because of (A1), we have
  $$\begin{aligned} \vdash \psi \rightarrow (\varphi \rightarrow \psi ) \end{aligned}$$
  and because of (d),
  $$\begin{aligned} \vdash (\psi \rightarrow (\varphi \rightarrow \psi ))\rightarrow ((\psi \vee (\varphi \rightarrow \psi ))\rightarrow (\varphi \rightarrow \psi )). \end{aligned}$$
  Now, (MP) implies
  $$\begin{aligned} \vdash (\psi \vee (\varphi \rightarrow \psi ))\rightarrow (\varphi \rightarrow \psi ). \end{aligned}$$
  By (a), we obtain
  $$\begin{aligned} \vdash ((\varphi \vee \psi )\rightarrow \psi )\rightarrow (\varphi \rightarrow \psi ). \end{aligned}$$
- According to (i), we have
  $$\begin{aligned} (\psi \vee \varphi )\rightarrow \varphi \leftrightarrow \psi \rightarrow \varphi \end{aligned}$$
  and according to (f),
  $$\begin{aligned} \varphi \rightarrow (\psi \vee \varphi )\vdash \lnot \varphi \rightarrow \lnot (\psi \vee \varphi )\leftrightarrow (\psi \vee \varphi )\rightarrow \varphi . \end{aligned}$$
  But
  $$\begin{aligned} \vdash \varphi \rightarrow (\psi \vee \varphi ) \end{aligned}$$
  holds because of (A1). Now, (j) follows from (a).

$$\square $$

## Algebraic semantics

For a class ${\mathcal {K}}$ of ${\mathcal {L}}$-algebras over a language ${\mathcal {L}}$, consider the relation $\models _{\mathcal {K}}$ that holds between a set $\Sigma$ of identities and a single identity $\varphi \approx \psi$ if every interpretation of $\varphi \approx \psi$ in a member of ${\mathcal {K}}$ holds provided each identity in $\Sigma$ holds under the same interpretation. In this case, we say that $\varphi \approx \psi$ is a ${\mathcal {K}}$-*consequence* of $\Sigma$. The relation $\models _{\mathcal {K}}$ is called the *semantic equational consequence relation* determined by ${\mathcal {K}}$.

Given a deductive system $({\mathcal {L}},\vdash _L)$ over a language ${\mathcal {L}}$, a class ${\mathcal {K}}$ of ${\mathcal {L}}$-algebras is called an *algebraic semantics* for $({\mathcal {L}},\vdash _L)$ if $\vdash _L$ can be interpreted in $\models _{\mathcal {K}}$ in the following sense: There exists a finite system $\delta _i(p)\approx \varepsilon _i(p)$, ($\delta (\varphi )\approx \varepsilon (\varphi )$, in brief) of identities with a single variable *p* such that for all $\Gamma \cup \{\varphi \}\subseteq {\mathcal {F}}m$,

$$\begin{aligned} \Gamma \vdash _L\varphi \Leftrightarrow \{\delta (\psi )\approx \varepsilon (\psi ),\psi \in \Gamma \}\models _{\mathcal {K}}\delta (\varphi )\approx \varepsilon (\varphi ). \end{aligned}$$

Then, $\delta _i\approx \varepsilon _i$ are called *defining identities* for $({\mathcal {L}},\vdash _L)$ and ${\mathcal {K}}$.

${\mathcal {K}}$ is said to be *equivalent* to $({\mathcal {L}},\vdash _L)$ if there exists a finite system $\Delta (p,q)$ of formulas with two variables *p*, *q* such that for every identity $\varphi \approx \psi$,where $\varphi \Delta \psi$ means just $\Delta (\varphi ,\psi )$ and  is an abbreviation for the conjunction $\Gamma \models _{\mathcal {K}}\Delta$ and $\Delta \models _{\mathcal {K}}\Gamma$.

According to Rasiowa (1974) and Rasiowa and Sikorski (1970), a *standard system of implicative extensional propositional calculus* (SIC, for short) is a deductive system $({\mathcal {L}},\vdash _L)$ satisfying the following conditions:

- The language ${\mathcal {L}}$ contains a finite number of connectives of rank 0, 1 and 2 and none of higher rank,
- ${\mathcal {L}}$ contains a binary connective $\rightarrow$ for which the following theorems and derived inference rules hold:
  $$\begin{aligned}&\vdash \varphi \rightarrow \varphi , \\&\varphi ,\varphi \rightarrow \psi \vdash \psi , \\&\varphi \rightarrow \psi ,\psi \rightarrow \chi \vdash \varphi \rightarrow \chi , \\&\varphi \rightarrow \psi ,\psi \rightarrow \varphi \vdash P(\varphi ) \rightarrow P(\psi ) \\&\qquad \text { for every unary }P\in {\mathcal {L}}, \\&\varphi \rightarrow \psi ,\psi \rightarrow \varphi ,\chi \rightarrow \lambda , \\&\lambda \rightarrow \chi \vdash Q(\varphi ,\chi )\rightarrow Q(\psi ,\lambda )\text { for every binary }Q\in {\mathcal {L}}. \end{aligned}$$

As one can immediately see, the system $({\mathcal {L}},\vdash _{\mathrm{LEA}})$ fulfils all the properties of SIC. Indeed, there is no unary connective in our language, and we have the only binary connective $\rightarrow$. Now, the first property is (c) of Theorem 3.1, the second is (MP) and the third is (a) from Theorem 3.1. To show the fifth property, we have $\varphi \rightarrow \psi \vdash (\psi \rightarrow \chi )\rightarrow (\phi \rightarrow \chi )$ by (Sf), and $\chi \rightarrow \lambda ,\lambda \rightarrow \chi \vdash (\psi \rightarrow \chi )\rightarrow (\psi \rightarrow \lambda )$ by (WPf). The rest then follows again by (a) of Theorem 3.1.

Moreover, taking $\varepsilon (p)=p$, $\delta (p)=p\rightarrow p$ and $\Delta (p,q)=\{p\rightarrow q,q\rightarrow p\}$, it is known (see Blok and Pigozzi 1989) that every SIC has an equivalent algebraic semantics with the defining identity $\delta \approx \varepsilon$ and with the set $\Delta$ as an equivalence system. As a consequence, we obtain

### Proposition 4.1

The logic $({\mathcal {L}},\vdash _{\mathrm{LEA}})$ is algebraizable with equivalence formulas $\Delta =\{p\rightarrow q,q\rightarrow p\}$ and the defining identity $p\approx p\rightarrow p$.

In order to show that LEA is an equivalent algebraic semantics for $({\mathcal {L}},\vdash _{\mathrm{LEA}})$, we use the following statement (see Blok and Pigozzi 1989, Theorem 2.17).

### Proposition 4.2

Let $({\mathcal {L}},\vdash _L)$ be a deductive system given by a set of axioms Ax and a set of inference rules Ir. Assume $({\mathcal {L}},\vdash _L)$ is algebraizable with equivalence formulas $\Delta$ and defining identities $\delta \approx \varepsilon$. Then, the unique equivalent semantics for $({\mathcal {L}},\vdash _L)$ is axiomatized by the identities

- $$\delta (\varphi )\approx \varepsilon (\varphi )$$ for each $$\varphi \in \mathrm{Ax}$$,
- $$\delta (p\Delta p)\approx \varepsilon (p\Delta p)$$

together with the quasiidentities

- $$\delta (\psi _0)\approx \varepsilon (\psi _0)\wedge \cdots \wedge \delta (\psi _{n-1})\approx \varepsilon (\psi _{n-1})\Rightarrow \delta (\varphi )\approx \varepsilon (\varphi )$$ for each $$\psi _0,\ldots ,\psi _{n-1}\vdash \varphi \in \mathrm{Ir}$$,
- $$\delta (p\Delta q)\approx \varepsilon (p\Delta q)\Rightarrow p\approx q$$.

Taking into account that $\vdash \varphi \rightarrow \varphi$ in $({\mathcal {L}},\vdash _{\mathrm{LEA}})$, we have $p\rightarrow p\approx 1$, i.e., $\varepsilon (p)=1$. Applying the previous proposition, we are going to prove the following.

### Theorem 4.3

The equivalent algebraic semantics for $({\mathcal {L}},f\vdash _{\mathrm{LEA}})$ is axiomatized by the following identities and quasiidentities:

1. $$x\rightarrow (y\rightarrow x)\approx 1$$,
2. $$((x\rightarrow y)\rightarrow y)\rightarrow ((y\rightarrow x)\rightarrow x)\approx 1$$,
3. $$0\rightarrow x\approx 1$$,
4. $$x\rightarrow x\approx 1$$,
5. $$x=x\rightarrow y=1\Rightarrow y=1$$,
6. $$x\rightarrow y=1\Rightarrow (y\rightarrow z)\rightarrow (x\rightarrow z)=1$$,
7. $$x\rightarrow y=1\Rightarrow (x'\rightarrow y')\rightarrow (y\rightarrow x)=1$$,
8. $$x\rightarrow y=y\rightarrow x=1\Rightarrow x=y$$,
9. $$x\rightarrow y'=(x'\rightarrow y)\rightarrow z'=1\Rightarrow ((x'\rightarrow y)'\rightarrow z)\rightarrow (x'\rightarrow (y'\rightarrow z))=1$$.

This system just corresponds to the quasivariety of lattice effect implication algebras.

### Proof

It can be immediately seen that any lattice effect implication algebra fulfills the above axioms. We will prove the converse, i.e., the properties listed above yield the conditions of Definition 2.3.

(i) The first two identities of (i) are just (3) and (4), and the remaining one follows from (4) and (1).
(ii) This is just (8).
(iii) Assume $x\rightarrow y=y\rightarrow z=1$. Then, according to (6) we have $(y\rightarrow z)\rightarrow (x\rightarrow z)=1$ which according to (5) yields $x\rightarrow z=1$.
  (i)–(iii) show that the relation $\le$ defined by $x\le y$ if and only if $x\rightarrow y=1$ is a partial order relation with smallest element 0 and greatest element 1.
(iv) This follows from (6).
(vi) According to (1) and (2), we have $x\le (y\rightarrow x)\rightarrow x\le (x\rightarrow y)\rightarrow y$.
(vii) According to (1), we have $y\le (x\rightarrow y)\rightarrow y$.
(v) We have
  $$\begin{aligned} (1\rightarrow x)\rightarrow x\approx & {} ((0\rightarrow x)\rightarrow x)\rightarrow x \\\approx & {} (x\rightarrow (0\rightarrow x))\rightarrow (0\rightarrow x) \\\approx & {} 1\rightarrow 1\approx 1 \end{aligned}$$
  according to (3), (2), (1) and (4) and therefore $x\le (0\rightarrow x)\rightarrow x=1\rightarrow x\le x$ according to (1) and (3), i.e., $1\rightarrow x\approx x$. Now,
  $$\begin{aligned} x''\approx (x\rightarrow 0)\rightarrow 0\approx (0\rightarrow x)\rightarrow x\approx 1\rightarrow x\approx x \end{aligned}$$
  according to (2) and (3).
(viii) Assume $x\le z$ and $y\le z$. Then, according to (6) and (2) we have $z\rightarrow y\le x\rightarrow y$ and
  $$\begin{aligned} (x\rightarrow y)\rightarrow y\le & {} (z\rightarrow y)\rightarrow y \\= & {} (y\rightarrow z)\rightarrow z=1\rightarrow z=z. \end{aligned}$$
(ix) Assume $x\le y$. Then, $y'\le x'$ according to (6) and
  $$\begin{aligned} y\rightarrow x=y''\rightarrow x''\le x'\rightarrow y'\le y\rightarrow x \end{aligned}$$
  according to (v) and (7).
(x) Assume $x\rightarrow y'=(x'\rightarrow y)\rightarrow z'=1$, i.e., $x\le y'$ and $x'\rightarrow y\le z'$. We know by (1) that $y\le x'\rightarrow y$, thus $y\le z'$. Now, we can apply (7) and (v) to obtain $y'\rightarrow z=z'\rightarrow y$. Using (6), $x'\rightarrow y\le z'$ yields $z'\rightarrow y\le (x'\rightarrow y)\rightarrow y=x'\vee y=x'$. This shows that $y'\rightarrow z\le x'$ which due to (iv) gives $x\le (y'\rightarrow z)'$. The converse implication follows by interchanging the elements *x* and *z*. Finally, under the above assumptions we have by (9) $(x'\rightarrow y)'\rightarrow z\le x'\rightarrow (y'\rightarrow z)$. Again, by interchanging the elements *x* and *z* we obtain the converse inequality and thus equality.
(xi) Applying (vii), (ix), (2) and (1), we obtain
  $$\begin{aligned} y'\rightarrow ((x\rightarrow y)\rightarrow y)'&\approx ((x\rightarrow y)\rightarrow y)\rightarrow y \\&\approx (y\rightarrow (x\rightarrow y))\rightarrow (x\rightarrow y) \\&\approx 1\rightarrow (x\rightarrow y)\approx x\rightarrow y. \end{aligned}$$
(xii) This is just (1).$\square$

We conclude that, using the equivalence between lattice effect algebras and lattice effect implication algebras and Theorem 4.3, system ${\mathbf {A}}$ is an algebraic axiomatization of the logic of lattice effect algebras.

## Effect implication algebras

Although lattice effect algebras are more feasible for some kinds of algebraic investigation, effect algebras which need not be lattice-ordered play a more important role in algebraic axiomatization of the logic of quantum mechanics. Hence, it is a question if also in this case we are able to derive some semantics, axioms and rules for a Gentzen-type axiomatization in a way similar to that for lattice-ordered effect algebras.

The first question is how to define the connective implication in not lattice-ordered effect algebras. In this section, we show that this can be done successfully in the case when the effect algebra $(E,+,{}',0)$ in question is finite, or, more generally, if for every $x,y\in E$ there exist maximal elements in the lower cone $L(x',y')$.

In the following, let ${\mathbf {E}}=(E,+,{}',0,1)$ be an effect algebra with induced order $\le$ such that $(E,\le )$ satisfies the ascending chain condition (shortly, ACC). This condition says that in $(E,\le )$ there do not exist infinite ascending chains. Hence, if $(E,\le )$ satisfies the ACC, then every non-empty subset of *E* has at least one maximal element. In particular, this is true in case of finite *E*.

Now, let $a,b\in E$ and $A,B\subseteq E$. Here and in the following,

$$\begin{aligned}&\text {Max}A\text { denotes the set of all maximal elements of }(A,\le ), \\&A+B:=\{x+y\mid x\in A,y\in B\}, \\&A\le B\text { means }x\le y\text { for all }x\in A\text { and }y\in B, \\&\text {we often identify }\{a\}\text { with }a. \end{aligned}$$

Moreover, we define

$$\begin{aligned} a\rightarrow b&:=b+\text {Max}L(a',b'), \\ a\rightarrow A&:=\bigcup _{x\in A}(a\rightarrow x). \end{aligned}$$

Since $(E,\le )$ satisfies the ACC, we have $x\rightarrow y\ne \emptyset$ for all $x,y\in E$. Moreover, if ${\mathbf {E}}$ is a lattice effect algebra, then the definition of $\rightarrow$ coincides with the original natural implication $\rightarrow$ since then

$$\begin{aligned} x\rightarrow y= & {} y+\text {Max}L(x',y')=y+\text {Max}L(x'\wedge y') \\= & {} y+(x'\wedge y')=y+(x\vee y)' \end{aligned}$$

for all $$x,y\in E$$.

Since every element of $\text {Max}L(x',y')$ is less than or equal to $y'$, $y+\text {Max}L(x',y')$ is defined for each $x,y\in E$. It is worth noticing that now $x\rightarrow y$ need not be a single element of *E*, but a non-void subset of *E*. Hence, one cannot expect that such an implication will satisfy the same properties as the implication in lattice effect algebras. On the other hand, it was already successfully used by the first and third author in Chajda and Länger (submitted) in order to show that for the implication defined in this way the effect algebra in question can be organized in an operator residuated structure.

Although not all the conditions of Theorems 2.1 and 2.2 are valid for effect algebras which need not be lattice-ordered, we still can prove several characteristic properties.

### Theorem 5.1

Let $(E,+,{}',0,1)$ be an effect algebra satisfying the ACC and $a,b\in E$. Then,

(i) $a\le b$ if and only if $a\rightarrow b=1$,
(ii) If $a\le b'$, then $a+b=a'\rightarrow b$,
(iii) If $a\ge b$, then $a\rightarrow b=a'+b$,
(iv) If $a\ge b$, then $a\rightarrow b=b'\rightarrow a'$,
(v) $$a\rightarrow 0=a'$$,
(vi) $$1\rightarrow a=a$$,
(vii) $$b'\rightarrow \text {Max}L(a',b')=a\rightarrow b$$.

### Proof

(i) The following are equivalent:
  $$\begin{aligned} a&\le b, \\ b'&\le a', \\ \text {Max}L(a',b')&=b', \\ b+\text {Max}L(a',b')&=1, \\ a\rightarrow b&=1. \end{aligned}$$
(ii) If $a\le b'$, then $a'\rightarrow b=b+\text {Max}L(a,b')=b+\text {Max}L(a)=b+a=a+b$.
(iii) If $a\ge b$, then $a\rightarrow b=a''\rightarrow b=a'+b$.
(iv) If $a\ge b$, then $a\rightarrow b=a'+b=b+a'=b''+a'=b'\rightarrow a'$.
(v) We have $$a\rightarrow 0=0+\text {Max}L(a',0')=0+\text {Max}L(a')=0+a'=a'$$.
(vi) We have $1\rightarrow a=a+\text {Max}L(1',a')=a+\text {Max}L(0)=a+0=a$.
(vii) We have
  $$\begin{aligned} b'\rightarrow \text {Max}L(a',b')&=\{b'\rightarrow x\mid x\in \text {Max}L(a',b')\} \\&=\{b''+x\mid x\in \text {Max}L(a',b')\} \\&=\{b+x\mid x\in \text {Max}L(a',b')\} \\&=b+\text {Max}L(a',b')=a\rightarrow b. \end{aligned}$$

$$\square $$

Summarizing all what was stated for implication in effect algebras, we can introduce the following concept.

### Definition 5.2

An *effect implication algebra* is an algebra $(I,\rightarrow ,0)$ of type (2, 0) satisfying the following conditions for all $x,y,z\in I$ (we abbreviate $x\rightarrow 0$ by $x'$ and $0'$ by 1):

(i) $$0\rightarrow x\approx x\rightarrow x\approx x\rightarrow 1\approx 1$$,
(ii) If $x\rightarrow y=y\rightarrow x=1$, then $x=y$,
(iii) If $x\rightarrow y=y\rightarrow z=1$, then $x\rightarrow z=1$,
(iv) If $x\rightarrow y=1$, then $y'\rightarrow x'=1$,
(v) $$x''\approx x$$,
(vi) If $x\rightarrow y=1$, then $y\rightarrow x=x'\rightarrow y'$,
(vii) $x\rightarrow y'=(x'\rightarrow y)\rightarrow z'=1$ if and only if $y\rightarrow z'=x\rightarrow (y'\rightarrow z)'=1$, and in this case $(x'\rightarrow y)'\rightarrow z=x'\rightarrow (y'\rightarrow z)$,
(viii) $$x\rightarrow (y\rightarrow x)\approx 1$$

and satisfying the condition that there does not exist an infinite sequence $a_1,a_2,a_3,\ldots$ of pairwise distinct elements of *I* satisfying $a_n\rightarrow a_{n+1}=1$ for all positive integers *n*.

Of course, every finite effect implication algebra satisfies trivially the last condition of Definition 5.2.

We are going to show that every effect algebra satisfying the ACC induces an effect implication algebra and vice versa.

### Theorem 5.3

Let ${\mathbf {E}}=(E,+,{}',0,1)$ be an effect algebra satisfying the ACC with induced order $\le$ and put

$$\begin{aligned} x\rightarrow y:=y+\text {Max}L(x',y') \end{aligned}$$

for all $x,y\in E$. Then, ${\mathbb {I}}({\mathbf {E}}):=(E,\rightarrow ,0)$ is an effect implication algebra. Moreover,

$$\begin{aligned} x\le y\text { if and only if }x\rightarrow y=1. \end{aligned}$$

### Proof

The proof follows from Theorem 5.1. More precisely, (i)–(iii) follow from the fact that $(E,\le ,0,1)$ is a bounded poset, (iv) follows from the fact that $'$ is antitone on $(E,\le )$, (v) follows from the fact that $'$ is an involution, (vi) follows from (E1), (vii) from (E2), and (viii) from (i) of Theorem 5.1 and from the definition of $\rightarrow$. $\square$

Although $x\rightarrow y$ need not be a single element of the corresponding effect algebra ${\mathbf {E}}$, we are able to prove that an effect implication algebra can be converted into an effect algebra where the partial operation $+$ is defined in the standard way.

### Theorem 5.4

Let ${\mathbf {I}}=(I,\rightarrow ,0)$ be an effect implication algebra and put

$$\begin{aligned} x'&:=x\rightarrow 0, \\ 1&:=0', \\ x+y&:=x'\rightarrow y\text { if and only if }x\rightarrow y'=1 \end{aligned}$$

for all $x,y\in E$. Then, ${\mathbb {E}}({\mathbf {I}}):=(I,+,{}',0,1)$ is an effect algebra satisfying the ACC with induced order $\le$ where

$$\begin{aligned} x\le y\text { if and only if }x\rightarrow y=1. \end{aligned}$$

### Proof

Let $$a,b,c\in I$$. Define

$$\begin{aligned} x\le y\text { if and only if }x\rightarrow y=1 \end{aligned}$$

for all $x,y\in I$. Then, due to (i)–(v) of Definition 5.2, $(I,\le ,{}',0,1)$ is a bounded poset with an antitone involution. If $a+b$ is defined, then $a\le b'$ and hence $b\le a'$, i.e., $b+a$ is defined and

$$\begin{aligned} a+b=a'\rightarrow b=b'\rightarrow a=b+a \end{aligned}$$

according to (vi) of Definition 5.2. This shows (E1). Now, $a+b$ is defined if and only if $a\le b'$, and in this case $a+b=a'\rightarrow b$. If $a+b$ is defined, then $(a+b)+c$ is defined if and only if $a+b\le c'$, and in this case

$$\begin{aligned} (a+b)+c=(a+b)'\rightarrow c=(a'\rightarrow b)'\rightarrow c. \end{aligned}$$

Hence, $(a+b)+c$ is defined if and only if both $a\le b'$ and $a'\rightarrow b\le c'$. On the other hand, $b+c$ is defined if and only if $b\le c'$, and in this case $b+c=b'\rightarrow c$. If $b+c$ is defined, then $a+(b+c)$ is defined if and only if $a\le (b+c)'$, and in this case

$$\begin{aligned} a+(b+c)=a'\rightarrow (b+c)=a'\rightarrow (b'\rightarrow c). \end{aligned}$$

Hence, $a+(b+c)$ is defined if and only if both $b\le c'$ and $a\le (b'\rightarrow c)'$. According to (vii) of Definition 5.2, (E2) holds. Now, the following are equivalent:

$$\begin{aligned}&a+b=1, \\&a\le b'\text { and }a'\rightarrow b=1, \\&b\le a'\text { and }a'\le b, \\&b=a'. \end{aligned}$$

This shows (E3). If $1+a$ is defined, then $1\le a'$ whence $a'=1$, i.e., $a=0$ showing (E4). Hence, ${\mathbb {E}}({\mathbf {I}})$ is an effect algebra satisfying the ACC whose induced order coincides with $\le$. $\square$

The following theorem shows that the correspondence between an effect algebra satisfying the ACC and its corresponding effect implication algebra is almost one-to-one.

### Theorem 5.5

The following hold:

(i) If ${\mathbf {E}}$ is an effect algebra satisfying the ACC, then ${\mathbb {E}}({\mathbb {I}}({\mathbf {E}}))={\mathbf {E}}$,
(ii) If ${\mathbf {I}}$ is an effect implication algebra satisfying the identity
  (10) $$x\rightarrow y\approx y'\rightarrow \text {Max}L(x',y')$$,
  then $${\mathbb {I}}({\mathbb {E}}({\mathbf {I}}))={\mathbf {I}}$$.

### Proof

Let ${\mathbf {E}}=(E,+,{}',0,1)$ be an effect algebra satisfying the ACC, put ${\mathbb {I}}({\mathbf {E}})=(E,\rightarrow ,0)$ and ${\mathbb {E}}({\mathbb {I}}({\mathbf {E}}))=(E,\oplus ,{}^*,0,e)$ and let $a,b\in E$. Then, $a\oplus b$ is defined if and only if so is $a+b$ since both are equivalent to $a\le b'$, and in this case

$$\begin{aligned} a\oplus b&=a'\rightarrow b=a+b, \\ a^*&=a\rightarrow 0=a', \\ e&=0\rightarrow 0=0'=1 \end{aligned}$$

according to Theorem 5.1 showing ${\mathbb {E}}({\mathbb {I}}({\mathbf {E}}))={\mathbf {E}}$. Conversely, let ${\mathbf {I}}=(I,\rightarrow ,0)$ be an effect implication algebra satisfying identity (10), put ${\mathbb {E}}({\mathbf {I}})=(I,+,{}',0,1)$ and ${\mathbb {I}}({\mathbb {E}}({\mathbf {I}}))=(I,\Rightarrow ,0)$ and let $c,d\in I$. Then,

$$\begin{aligned} c\Rightarrow d&=d+\text {Max}L(c',d')=\{d+x\mid x\in \text {Max}(c',d')\} \\&=\{d'\rightarrow x\mid x\in \text {Max}(c',d')\} \\&=d'\rightarrow \text {Max}L(c',d')=c\rightarrow d \end{aligned}$$

according to (10). This shows ${\mathbb {I}}({\mathbb {E}}({\mathbf {I}}))={\mathbf {I}}$. $\square$

Theorem 5.5 (i) says that every effect algebra satisfying the ACC is fully determined by its derived effect implication algebra. This enables us to set up the semantics and axiomatization for finite effect algebras which need not be lattice-ordered in a way similar to that for lattice effect algebras.

## The logic of finite effect algebras

In this section, we use the same general theory taken from Blok and Pigozzi (1989) as in Sects. 3 and 4. Hence, we need not repeat all what was said in the beginning of these two sections. All what was described in Propositions 4.1 and 4.2 remains valid also here with the same equivalence system.

By the propositional logic $L_{\mathrm{EA}}$ in a language ${\mathcal {L}}=\{\rightarrow ,0\}$ ($\rightarrow$ is of rank 2, 0 is of rank 0), we understand a consequence relation $\vdash _{\mathrm{EA}}$ (or $\vdash$, in brief) satisfying the axioms

1. $$\vdash \varphi \rightarrow (\psi \rightarrow \varphi )$$,
2. $$\vdash \varphi \rightarrow \varphi $$,
3. $$\vdash \varphi \leftrightarrow (\varphi \rightarrow 0)\rightarrow 0$$,
4. $$\vdash 0\rightarrow \varphi $$

and the rules

(MP) $$\varphi ,\varphi \rightarrow \psi \vdash \psi $$,
(Sf) $$\varphi \rightarrow \psi \vdash (\psi \rightarrow \chi )\rightarrow (\varphi \rightarrow \chi )$$,
(WPf) $$\varphi \rightarrow \psi ,\psi \rightarrow \varphi \vdash (\chi \rightarrow \varphi )\rightarrow (\chi \rightarrow \psi )$$,
(R1) $$\varphi \rightarrow \psi \vdash (\lnot \varphi \rightarrow \lnot \psi )\rightarrow (\psi \rightarrow \varphi )$$,
(R2) $$\varphi \rightarrow \lnot \psi ,(\lnot \varphi \rightarrow \psi )\rightarrow \lnot \chi \vdash (\lnot (\lnot \varphi \rightarrow \psi )\rightarrow \chi )\rightarrow (\lnot \varphi \rightarrow (\lnot \psi \rightarrow \chi ))$$,

where $$\lnot \varphi :=\varphi \rightarrow 0$$ and $$1:=\lnot 0=0\rightarrow 0$$.

This axiom system will be referred to as system ${\mathbf {B}}$. We can prove the following consequences of ${\mathbf {B}}$.

### Theorem 6.1

In the propositional logic $L_{\mathrm{EA}}$, the following are provable:

- $$\varphi \rightarrow \psi ,\psi \rightarrow \chi \vdash \varphi \rightarrow \chi $$,
- $$\vdash \varphi \rightarrow 1$$,
- $$\vdash \lnot \lnot \varphi \leftrightarrow \varphi $$,
- $$\varphi \rightarrow \psi \vdash \lnot \varphi \rightarrow \lnot \psi \leftrightarrow \psi \rightarrow \varphi $$.

### Proof

- This follows from (Sf) and (MP).
- According to (B1),
  $$\begin{aligned} \vdash 1\rightarrow (\varphi \rightarrow 1) \end{aligned}$$
  and according to (B4)
  $$\begin{aligned} \vdash 0\rightarrow 0, \end{aligned}$$
  i.e.,
  $$\begin{aligned} \vdash 1. \end{aligned}$$
  Thus, applying (MP) we conclude
  $$\begin{aligned} \vdash \varphi \rightarrow 1. \end{aligned}$$
- This is (B3).
- According to (R1), we have
  $$\begin{aligned} \varphi \rightarrow \psi \vdash (\lnot \varphi \rightarrow \lnot \psi )\rightarrow (\psi \rightarrow \varphi ). \end{aligned}$$
  Moreover,
  $$\begin{aligned} \vdash (\psi \rightarrow \varphi )\rightarrow (\lnot \varphi \rightarrow \lnot \psi ) \end{aligned}$$
  according to (Sf).

$$\square $$

In what follows, we show that this algebraic semantics is just that for algebras satisfying conditions (i)–(viii) of Definition 5.2, i.e., for finite effect implication algebras. Similar to the lattice case, the system $({\mathcal {L}},\vdash _{\mathrm{EA}})$ fulfills the properties of SIC. Again, applying Proposition 4.2, we are going to show the following theorem:

### Theorem 6.2

The equivalent algebraic semantics for $({\mathcal {L}},\vdash _{\mathrm{EA}})$ is axiomatized by the following identities and quasiidentities (we abbreviate $x\rightarrow 0$ by $x'$ and $0'$ by 1):

1. $$x\rightarrow (y\rightarrow x)\approx 1$$,
2. $$x\rightarrow x''\approx 1$$ and $$x''\rightarrow x\approx 1$$,
3. $$0\rightarrow x\approx 1$$,
4. $$x\rightarrow x\approx 1$$,
5. $$x=x\rightarrow y=1\Rightarrow y=1$$,
6. $$x\rightarrow y=1\Rightarrow (y\rightarrow z)\rightarrow (x\rightarrow z)=1$$,
7. $$x\rightarrow y=1\Rightarrow (x'\rightarrow y')\rightarrow (y\rightarrow x)=1$$,
8. $$x\rightarrow y=y\rightarrow x=1\Rightarrow x=y$$,
9. $$x\rightarrow y'=(x'\rightarrow y)\rightarrow z'=1\Rightarrow ((x'\rightarrow y)'\rightarrow z)\rightarrow (x'\rightarrow (y'\rightarrow z))=1$$,
10. $$x\rightarrow y=1\Rightarrow (y\rightarrow x)\rightarrow x=y$$.

This system just corresponds to properties (i)–(viii) of Definition 5.2.

### Proof

We will show that the corresponding algebra satisfies the conditions listed in Definition 5.2.

(i) The first two identities of (i) are just (3) and (4), and the remaining one follows from (4) and (1).
(ii) This is just (8).
(iii) Assume $x\rightarrow y=y\rightarrow z=1$. Then, according to (6) we have $(y\rightarrow z)\rightarrow (x\rightarrow z)=1$ which according to (5) yields $x\rightarrow z=1$.
  (i)–(iii) show that the relation $\le$ defined by $x\le y$ if and only if $x\rightarrow y=1$ is a partial order relation with the smallest element 0 and greatest element 1.
(iv) This follows from (6).
(v) This follows from (2) and (8).
(vi) Assume $x\le y$. Then, $y'\le x'$ according to (6) and
  $$\begin{aligned} y\rightarrow x=y''\rightarrow x''\le x'\rightarrow y'\le y\rightarrow x \end{aligned}$$
  according to (v) and (7).
(vii) Assume $x\rightarrow y'=(x'\rightarrow y)\rightarrow z'=1$, i.e., $x\le y'$ and $x'\rightarrow y\le z'$. We know by (1) that $y\le x'\rightarrow y$, thus $y\le z'$. Now, we can apply (7) and (v) to obtain $y'\rightarrow z=z'\rightarrow y$. Using (6) and (10), $x'\rightarrow y\le z'$ yields $z'\rightarrow y\le (x'\rightarrow y)\rightarrow y=x'$. This shows that $y'\rightarrow z\le x'$ which due to (iv) gives $x\le (y'\rightarrow z)'$. The converse implication follows by interchanging the elements *x* and *z*. Finally, under the above assumptions we have by (9) $(x'\rightarrow y)'\rightarrow z\le x'\rightarrow (y'\rightarrow z)$. Again, by interchanging the elements *x* and *z* we obtain the converse inequality and thus equality.
(viii) This is just (1).

$$\square $$

Due to the correspondence described in Theorem 5.5, our propositional logic $L_{\mathrm{EA}}$, i.e., system ${\mathbf {B}}$, is an algebraic axiomatization in Gentzen style of the logic of finite effect algebras which need not be lattice-ordered. A possible extension to effect algebras satisfying ACC could make problems since the last condition of Definition 5.2 (mentioned after condition (viii)) equivalent to ACC cannot be easily described in our logic $L_{\mathrm{EA}}$.
